# Anti-inflammatory effect of *Gyeji-tang* in a chronic obstructive pulmonary disease mouse model induced by cigarette smoke and lipopolysaccharide

**DOI:** 10.1080/13880209.2022.2131841

**Published:** 2022-10-20

**Authors:** Eun Bok Baek, Yu Jin Kim, Jin-Hyung Rho, Eun-Ju Hong, Mee-Young Lee, Hyo-Jung Kwun

**Affiliations:** a Department of Veterinary Pathology, Collage of Veterinary Medicine, Chungnam National University, Daejeon, Korea; b Herbal Medicine Research Division, Korea Institute of Oriental Medicine, Daejeon, Korea

**Keywords:** COPD animal model, inflammation, airway remodelling

## Abstract

**Context:**

Chronic obstructive pulmonary disease (COPD) is a chronic inflammatory lung disease associated with respiratory symptoms and narrowing of airways. *Gyeji-tang* (GJT) is a traditional Asian medicine that has been used to relieve early-stage cold symptoms, headache, and chills.

**Objective:**

We examined the effect and potential molecular action mechanism of GJT in a mouse model of COPD induced by cigarette smoke (CS) plus lipopolysaccharide (LPS).

**Materials and methods:**

COPD was induced in C57BL/6J mice via daily exposure to CS for 1 h for 8 weeks and intranasal administration of LPS on weeks 1, 3, 5, and 7. GJT (100 or 200 mg/kg) or roflumilast (5 mg/kg) was administrated daily for the final 4 weeks of COPD induction.

**Results:**

Administration of GJT significantly suppressed the CS/LPS-induced increases in: the numbers of total cells and macrophages in bronchoalveolar lavage fluid; the expression levels of tumour necrosis factor-α, interleukin (IL)-6, IL-1β, and IL-8; the activities (phosphorylation) of nuclear factor kappa B and signal transducer and activator of transcription 3; and the expression levels of the structural remodelling markers, transforming growth factor beta, matrix metallopeptidase (MMP)-7, and MMP-9.

**Discussion and conclusions:**

These results demonstrate that GJT prevents the lung inflammation and airway remodelling induced by CS plus LPS exposure in mice, suggesting that GJT may have therapeutic potential for the treatment of COPD.

## Introduction

The inflammatory response is a natural defence mechanism through which the body responds to harmful stimuli. Leukocytes (e.g., dendritic cells and macrophages), adaptive immunity T cells, B cells, and non-immune cells (e.g., epithelial cells, endothelial cells, and fibroblasts) express immune pathogen-recognition receptors called toll-like receptors (TLRs) (Delneste et al. 2007). Upon pathogen recognition, proinflammatory mediators (e.g., cytokines, chemokines, and growth factors) are produced under regulation by transcription factors, such as nuclear factor kappa-light-chain-enhancer of activated B-cells (NF-κB) and mitogen-activated protein kinases (MAPKs) (Saklatvala et al. 2003). In addition, transcriptional regulator signal transducer and activator of transcription 3 (STAT3) plays a key role in regulating inflammation and immunity (Hillmer et al. 2016). Airway inflammation usually arises due to exposure to inhaled toxins, pollutants, irritants, and/or allergens. The airway epithelium is the first site of contact with inhaled agents (Moldoveanu et al. 2009). The cells of the airway epithelium secrete mucins and lysozyme, which non-specifically protect the respiratory tract from external attack. Cigarette smoke (CS) encompass innate immune, which release proinflammatory cytokines and chemokines, such as tumour necrosis factor (TNF)-α, interleukin (IL)-6, IL-1β, and IL-8 by airway epithelial cells and alveolar macrophages elicits the expression of adhesion molecules on endothelial cells and the recruitment of inflammatory macrophages to the lungs (Barnes 2016; Brusselle et al. 2011). These cytokines appear to amplify inflammation, in part through the activation of the transcription factor, NF-κB and STAT3, thereby leading to the increased expression of multiple inflammatory genes.

The airway inflammatory diseases include chronic obstructive pulmonary disease (COPD), which is generally initiated by CS. Persistent CS stimuli evoke a chronic inflammation response (Lane et al. 2010), and CS has been shown to directly trigger TLRs, leading to the -mediated production of TNF-α, IL-6, and IL-10 (Metcalfe et al. 2014). The pathophysiology of COPD is generally believed to involve the combined processes of peripheral airway inflammation and structural change-related airway narrowing (Bourdin et al. 2009).

*Gyeji-tang* (GJT) is a traditional Asian medicine that has been used to relieve early-stage cold symptoms, headache, and chills (Yoo et al. 2016). GJT is called Guizhi Tang in Chinese and Keishi-to in Japanese, and has been used as a traditional remedy in both cultures (Heo 2004). GJT consists of five herbs: *Cinnamomi Ramulus, Paeoniae Radix, Glycyrrhizae Radix* et Rhizoma*, Zingiberis Rhizoma Crudus*, and *Zizyphi Fructus*. The main component is *Cinnamomi Ramulus*, which decreases the inflammatory response in BV2 microglial cells (Yang et al. 2017). *Paeoniae Radix*, meanwhile, harbours monoterpene glycosides that exhibited anti-inflammatory and antioxidative activities in the Raw 264.7 murine macrophage cell line (Lim et al. 2019). *Glycyrrhizae Radix et Rhizoma* has been used in cosmetic and food ingredients (Pastorino et al. 2018). The hexane fraction of *Zingiberis Rhizoma Crudus* extract was reported to inhibit the production of nitric oxide and proinflammatory cytokines (Jung et al. 2009). *Zizyphi Fructus* is a major constituent of a Chinese anti-asthmatic herbal medicine, and was reported to enhance airway ciliary motility (Tamaoki et al. 1996). To date, no published study has examined the effect of GJT on COPD. Here, we investigated the protective effects of JGT against COPD using a CS and lipopolysaccharide (LPS)-induced mouse model of COPD.

## Materials and methods

### Preparation and analysis of GJT

GJT was provided by the Herbal Medicine Research Division, Korea Institute of Oriental Medicine (Daejeon, South Korea). It was extracted as previously described in detail (Yoo et al. 2016). The water extract of GJT was dissolved in methanol to a concentration of 5 mg/mL and filtered through a 0.45-µm syringe filter. The compounds in GJT were analysed using a Waters Alliance e2695 system-photodiode array detector (Waters Corp., Milford, MA, USA). Reference standards (purity > 98%) for gallic acid, albiflorin, paeoniflorin, liquiritin apioside, liquiritin, coumarin, benzoylpaeoniflorin, cinnamaldehyde, glycyrrhizin, and 6-gingerol were purchased from ChemFaces (Wuhan, China). Chromatographic separation was conducted on a Sunfire C_18_ column (4.6 × 250 mm, 5 µm, Waters) at 40 °C using mobile phases consisting of 0.1% (v/v) aqueous formic acid (FA, Merck, Darmstadt, Germany) (a) and 0.1% (v/v) FA in acetonitrile (b). The utilized gradients were as follows: 5–60% (b) for 0–30 min, 60–100% (b) for 30–40 min, and 100% (b) for 40–50 min. The injection volume and flow rate were set at 10 µL and 1 mL/min, respectively.

### Animal model of COPD induced by CS/LPS

Specific pathogen-free male C57BL/6J mice (7 weeks of age) were obtained from Orient Bio (Seongnam, South Korea). The animals were acclimatized for 7 days before the initiation of dosing. During acclimation, the experimental animals were kept under environmentally controlled conditions (22 °C ± 2 °C; relative humidity, 50% ± 5%; 12 h light/dark cycle) and provided standard rodent chow and sterilized tap water ad libitum. All animal experiment procedures followed accepted ethical procedures and were approved by the Animal Experimental Ethics Committee of Chungnam National University (Daejeon, South Korea). The mice were randomly divided into five subgroups and each group was consisted seven to eight animals as follows: (i) normal control (NC) group; (ii) CS exposure with LPS intranasal administration (CS/LPS) group; (iii) CS exposure with LPS administration plus roflumilast 5 mg/kg oral gavage (RO) group, which was used as a positive control group; (iv) CS exposure with LPS administration plus GJT 100 mg/kg oral gavage (GJT100) group; and (v) CS exposure with LPS administration plus GJT oral gavage 200 mg/kg (GJT200) group. For CS exposure, 3R4F research cigarettes were purchased from the Tobacco and Health Research Institute at the University of Kentucky (KY, USA), and mice in an exposure chamber were exposed to room air with or without CS from eight cigarettes for 1 h daily for 8 weeks. For LPS exposure, anesthetized mice were given 10 µg of LPS by intranasal instillation on weeks 1, 3, 5, and 7. Roflumilast, a selective phosphodiesterase 4 inhibitor that decreases systemic and pulmonary inflammation and improves disease symptoms in patients with COPD (Garnock-Jones 2015), was used as a positive control drug. Roflumilast and GJT were administrated by 5 days in a week oral gavage given 1 h prior to CS exposure for the last 4 weeks of the CS-exposure protocol.

### Collection of blood and bronchoalveolar lavage fluid

Mice were sacrificed 48 h after the last exposure to CS. Whole-blood samples were collected by cardiac puncture. Serum was isolated by centrifugation for 15 min at 13,000 rpm and stored at −70 °C until analysis. Bronchoalveolar lavage fluid (BALF) was obtained from the lungs using a tracheal cannula and three rounds of lavage performed by instilling and withdrawing 1.5 mL of phosphate-buffered saline (PBS). The obtained BALF was centrifuged and the supernatant aliquoted and stored at −70 °C until analysis. Cells were counted under a haemocytometer from at least five squares. BALF (100 μL) was placed on a slide, which was then centrifuged (200 *g*, 10 min, 4 °C) using a Cytospin system (Hanil Science Industrial, Seoul, Korea). The slides were dried, and the cells were fixed and stained using Diff-Quik Stain reagents (B4132-1A; IMEB Inc., IL, USA).

### Histopathological examination

Lungs fixed in 10% neutral buffered formalin were paraffin-embedded and sectioned at 4 μm, and the sections were placed on glass slides, deparaffinized, and subjected to haematoxylin and eosin (H&E) staining. Histological changes, including inflammatory cell infiltration and alteration in epithelial thickness, were scored from 0 (absent) to 4 (severe) as previously described (Gori et al. 2019). For analysis of collagen deposition in lung tissues, paraffin sections were stained with Sirius red and Masson’s trichrome, counterstained with Mayer’s haematoxylin, and analysed via light microscopy.

### Immunohistochemistry assay

Immunohistochemistry (IHC) was performed according to a previously published protocol (Chen et al. 2016). Anti-matrix metallopeptidase (MMP)-7 (diluted 200:1; Abcam, Cambridge, UK) was applied overnight as the primary antibody, the results were developed and visualized according to the protocol, and the positive area was calculated for lung tissue samples from at least five animals per group.

### RNA extraction and real-time PCR analysis of mRNA expression

Total RNA was extracted using an RNeasy mini kit (Qiagen, MD, USA). The concentration of RNA was determined based on the absorbance (A) at 260 nm, and the purity was evaluated by measuring the A260/A280 ratio. Complementary DNA was generated from total RNA 1 μg using a reverse transcription kit (Qiagen) according to the manufacturer’s instructions. Real-time PCR was performed in an Applied Biosystems 7500 Real-Time PCR System (Life Technologies, CA, USA) using the SYBR Green PCR Master Mix (Life Technologies) per the manufacturer’s instructions. The utilized PCR primers were as follows: TNF-α, 5′-GTCTGTGCCTCAGCCTCTTC-3′ (forward) and 5′-CCCATTTGGGAACTTCCCT-3′ (reverse); IL-6, 5′-TAGTCCTTCCTACCCCAACT-3′ (forward) and 5′-TTGGTCCTTAGCCACTCCTT-3′ (reverse); IL-1β, 5′-AGGACCCAAGCACCTTCTTT-3′ (forward) and 5′-AGACAGCACGAGGCATTTT-3′ (reverse); IL-8, 5′-GATTCACCTCAAGAACATCCAGA-3′ (forward) and 5′-GGACACCTTTTAGCATCTTTTGG-3′ (reverse); transforming growth factor beta (TGF-β), 5′-TTGCTTCAGCTCCACAGAGA-3′ (forward) and 5′-TGGTTGTAGAGGGCAAGGAC-3′ (reverse); MMP-7, 5′-GTTTTTGATGCTATTGCTGA-3′ (forward) and 5′-CCCACATTTGACGTCCAGTCCAGAG-3′ (reverse); MMP-9, 5′-ACGACATAGACGCCA TCCAGT-3′ (forward) and 5′-AGGTATAGTGGGACGACTGGG-3′ (reverse); α-SMA, 5′-TGCTGACAGAGGCACCACTGAA-3′ (forward) and 5′-CAGTTGTACGTCCAGAGGCATAG-3′ (reverse); and GAPDH, 5′-ACAGCAACAGGGTGGTGGAC-3′ (forward) and 5′-TTTGAGGGTGCAGCGAACTT-3′ (reverse). The real-time PCR results were analysed using the Applied Biosystems 7500 Real-Time PCR System software (Applied Biosystems, CA, USA), and the fold change in the cDNA expression of the target gene relative to the endogenous constitutive control (GAPDH) was calculated using the 2^–ΔΔCt^ method.

### Western blot analysis

Equal amounts of total lung proteins (30 μg) were resolved by 10% SDS-PAGE and transferred to nitrocellulose membranes at 350 mA for 2 h. The membranes were blocked for at least 30 min with PBS containing 0.05% Tween 20 (PBS-T) supplemented with 5% bovine serum albumin (BSA), and then incubated overnight at 4 °C with anti-phospho-NF-κB, anti-phospho-STAT3, anti-NF-κB, anti-STAT3, IκB-α (all from Cell Signalling Technology, MA, USA), or anti-β-actin (Sigma Aldrich, MO, USA). The membranes were washed thrice with PBS-T for 10 min, and then incubated for 2 h at room temperature with a horseradish peroxidase-conjugated secondary antibody. The membranes were again washed thrice with PBS-T, and then developed using an enhanced chemiluminescence kit (Thermo Scientific, MA, USA).

### Statistical analysis

Data are expressed as the mean ± standard deviation (SD). Statistical comparisons were made with one-way analysis of variance (ANOVA) using Graphpad prism 6.0 (GraphPad Software, San Diego, USA), followed by Dunnett’s multiple comparison test. *p* < 0.05 was considered statistically significant.

## Results

### HPLC analysis of GJT

**Figure 1.**
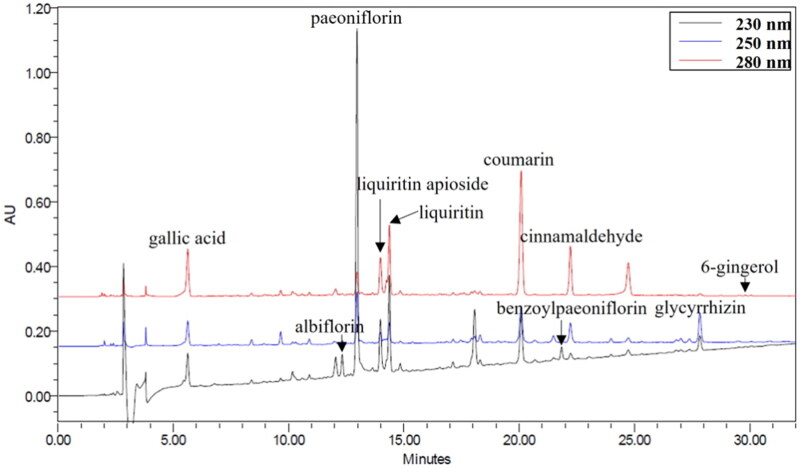
HPLC chromatograms of GJT. Extracted LC chromatograms for the phytochemicals constituents of GJT identified at 230, 250, and 280 nm. Shown are the chromatograms for albiflorin, paeoniflorin, benzoylpaeoniflorin, glycyrrhizin, 6-gingerol, gallic acid, liquiritin apioside, liquiritin, coumarin, and cinnamaldehyde.

Ten compounds in the water extract of GJT were identified by HPLC analysis and successfully separated within 30 min on a Sunfire C_18_ column (4.6 × 250 mm, 5 µm, Waters). The detection wavelengths were set at 230 nm for albiflorin, paeoniflorin, and benzoylpaeoniflorin, 250 nm for glycyrrhizin, and 280 nm for gallic acid, liquiritin apioside, liquiritin, coumarin, cinnamaldehyde, and 6-gingerol. The HPLC chromatograms obtained for the water extract of GJT at 230, 250, and 280 nm are shown in Figure 1. The regression equation, linear ranges, correlation coefficients (*r*^2^), the limits of detection (LOD) and quantification (LOQ), and contents for the 10 compounds are presented in Table 1. The contents of the 10 compounds in water extract of GJT ranged from 0.105 to 40.150 mg/g.

**Table 1.** Regression equation, linearity, LOD, LOQ, and content of the 10 compounds.

Compound	Linear range (µg/mL)	Regression equation (*y* = *ax* + *b*)^a^	Correlation coefficient (*r*^2^)	LOD^b^ (µg/mL)	LOQ^c^ (µg/mL)	Content (mg/g)
Gallic acid	1.5625–50	*y* = 25277*x* + 1312.2	1.0000	0.028	0.085	4.433 ± 0.007
Albiflorin	3.125–50	*y* = 13,082*x* + 338.22	1.0000	0.016	0.049	2.879 ± 0.040
Paeoniflorin	25–400	*y* = 15,539*x* − 453.53	1.0000	0.336	1.018	40.150 ± 0.206
Liquiritin apioside	1.5625–50	*y* = 13,752*x* + 2380.9	0.9999	0.007	0.022	5.647 ± 0.024
Liquiritin	1.5625–50	*y* = 17,419*x* + 4394	0.9999	0.042	0.127	6.921 ± 0.048
Coumarin	3.125–100	*y* = 42,702*x* + 29316	0.9998	0.085	0.256	7.242 ± 0.060
Benzoylpaeoniflorin	1.5625–25	*y* = 23,773*x* + 50.556	1.0000	0.016	0.048	1.175 ± 0.004
Cinnamaldehyde	3.125–100	*y* = 100,050*x* + 60,867	0.9998	0.091	0.276	1.344 ± 0.001
Glycyrrhizin	3.125–100	*y* = 6628.6*x* + 1223.4	1.0000	0.128	0.387	10.635 ± 0.028
6-Gingerol	0.195–6.25	*y* = 6780.2*x* − 14.113	1.0000	0.010	0.031	0.105 ± 0.001

^a^ *y*=*ax*+*b*, *y* means peak area and *x* means concentration (µg /mL).

^b^ LOD: 3.3 × [standard deviation (SD) of the response/slope of the calibration curve].

^c^ LOQ: 10 × (SD of the response/slope of the calibration curve).

### GJT prevents histological damage to lung tissues in a CS/LPS-induced mouse model of COPD

**Figure 2.**
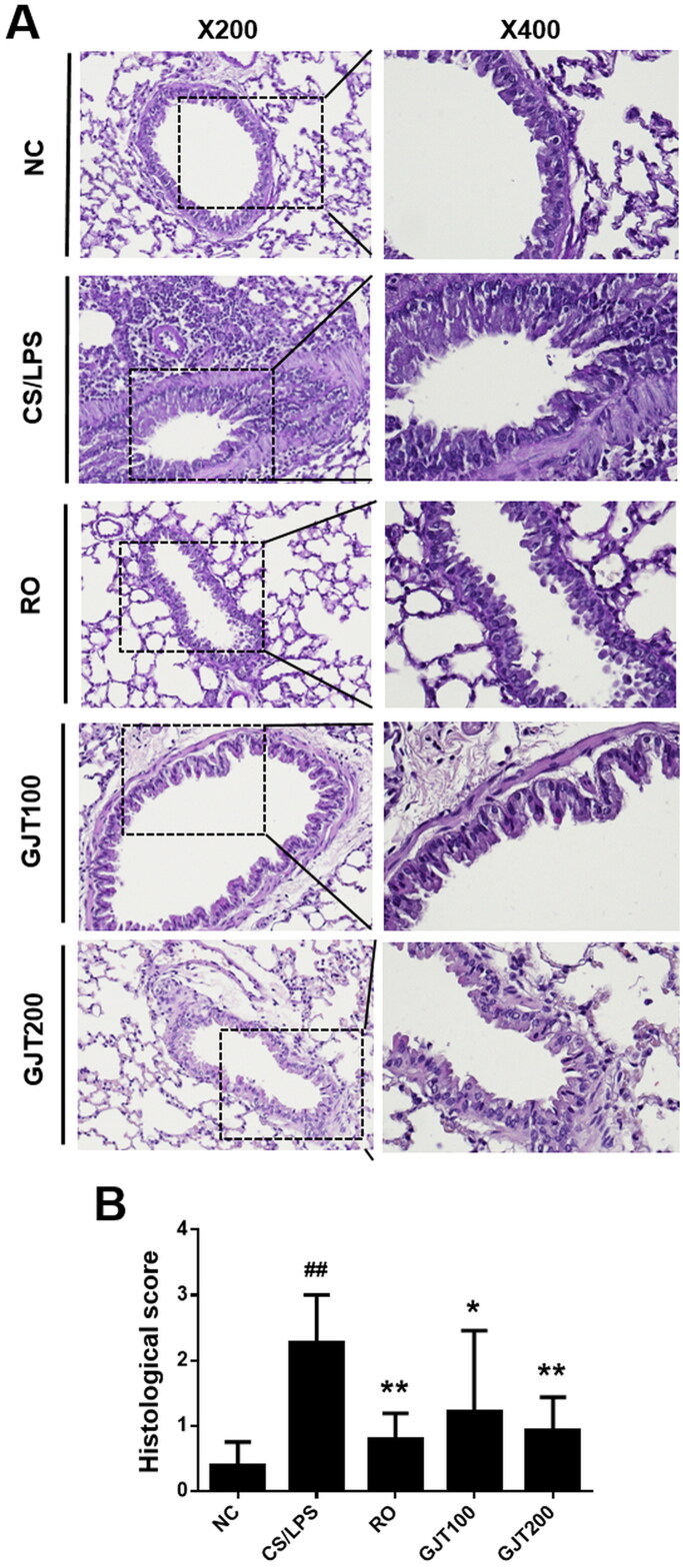
Effects of GJT on CS/LPS-induced damage in lung tissues, assessed by histological examination. (A) Representative H&E-stained lung tissues sectioned at 4-μm thickness. (B) Histological scoring for inflammation on a scale of 0 (absent) to 4 (severe). NC, normal control; CS/LPS, CS plus LPS-exposed; RO, roflumilast (5 mg/kg) administered prior to CS/LPS exposure; GJT100, GJT (100 mg/kg) administered prior to CS/LPS exposure; GJT200, GJT (200 mg/kg) administered prior to CS/LPS exposure. Results are presented as mean ± SD. ^#^*p* < 0.05 and ^##^*p* < 0.01 compared with the NC group; *p < 0.05 and **p < 0.01 compared with the CS/LPS group.

H&E staining was performed to examine pulmonary histopathological changes associated with GJT treatment. Lung sections from the NC group showed a normal bronchoalveolar structure with few inflammatory cells, whereas those from the CS/LPS group showed increases in inflammatory cell infiltration and airway wall thickness (Figure 2(A)). Notably, the tissues from the roflumilast- and GJT-treated groups showed reduced inflammatory cell accumulation and airway wall thickening compared with CS/LPS-exposed mice (Figure 2(A)). As shown in Figure 2(B), alveolar walls were thickened with decreased airspace and infiltration of inflammatory cells was increased in the CS/LPS group, whereas GJT treatment significantly reversed these effects. These results indicate that GJT significantly decreases the lung inflammation and injury triggered by CS plus LPS.

### GJT prevents the CS/LPS-induced influx of inflammatory cells into BALF

**Figure 3.**
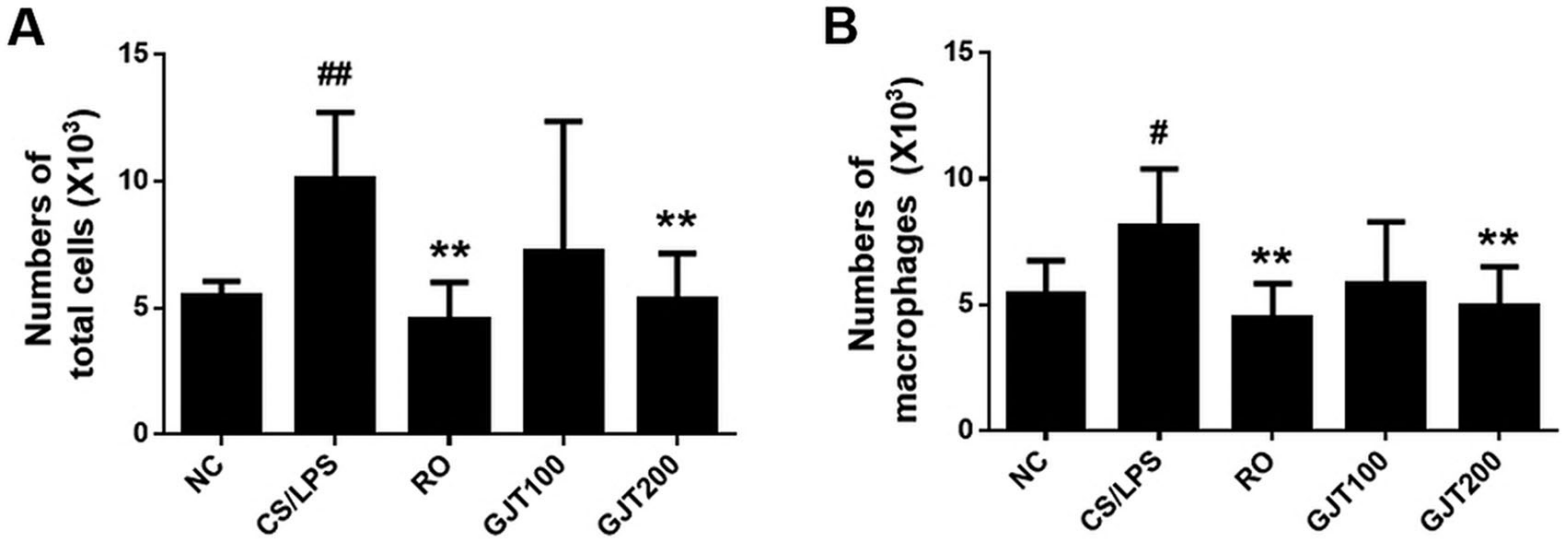
Effects of GJT on CS/LPS-induced recruitment of inflammatory cells into BALF. (A) Numbers of total cells in BALF. (B) Numbers of macrophages in BALF. NC, normal control; CS/LPS, CS plus LPS-exposed; RO, roflumilast (5 mg/kg) administered prior to CS/LPS exposure; GJT100, GJT (100 mg/kg) administered prior to CS/LPS exposure; GJT200, GJT (200 mg/kg) administered prior to CS/LPS exposure. Results are presented as mean ± SD. ^#^*p* < 0.05 and ^##^*p* < 0.01 compared with the NC group; *p < 0.05 and **p < 0.01 compared with the CS/LPS group.

We evaluated the effects of GJT on CS/LPS-induced pulmonary inflammation responses by monitoring inflammatory cell counts in BALF. The numbers of total inflammatory cells, lymphocytes, macrophages, eosinophils, and neutrophils in BALF were examined. We found that the numbers of total inflammatory cells and macrophages were significantly higher in BALF from the CS/LPS group compared with the NC group, and that roflumilast- or GJT-treated mice (200 mg/kg) showed lower numbers of total cells and macrophages in BALF compared with the CS/LPS group (Figure 3). These results indicate that GJT prevents the CS/LPS-induced recruitment of inflammatory cells into lung tissues.

### GJT attenuates CS/LPS-induced inflammatory cytokine production

**Figure 4.**
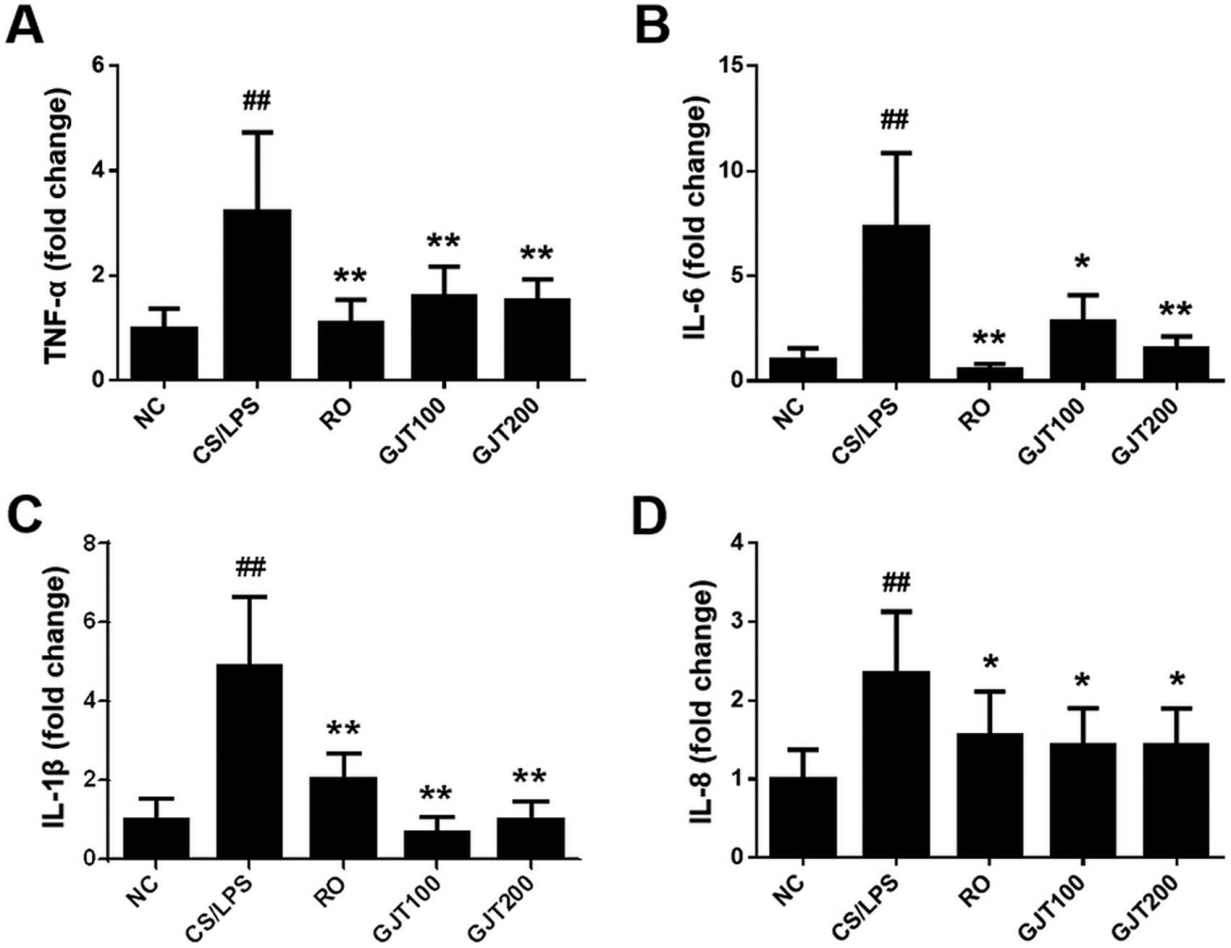
Effects of GJT on the relative expression levels of inflammatory cytokines in lung tissues of CS/LPS-treated mice. Relative mRNA expression levels of TNF-α (A), IL-6 (B), IL-1β (C), and IL-8 (D) in lung tissues. NC, normal control; CS/LPS, CS plus LPS-exposed; RO, roflumilast (5 mg/kg) administered prior to CS/LPS exposure; GJT100, GJT (100 mg/kg) administered prior to CS/LPS exposure; GJT200, GJT (200 mg/kg) administered prior to CS/LPS exposure. Results are presented as mean ± SD. ^#^*p* < 0.05, ^##^*p* < 0.01 compared with the NC group; *p < 0.05, **p < 0.01 compared with the CS/LPS group.

We evaluated the effect of GJT on the expression levels of several inflammation-related cytokines and chemokines in lung tissues. We found that CS/LPS treatment increased the relative mRNA expression levels of TNF-α, IL-6, L-1β, and IL-8 and that GJT or roflumilast significantly attenuated these upregulations (Figure 4). Our findings suggest that GJT significantly alleviates the inflammatory cytokine production induced by CS/LPS.

### GJT prevents CS/LPS-induced NF-κB and STAT3 signalling

**Figure 5.**
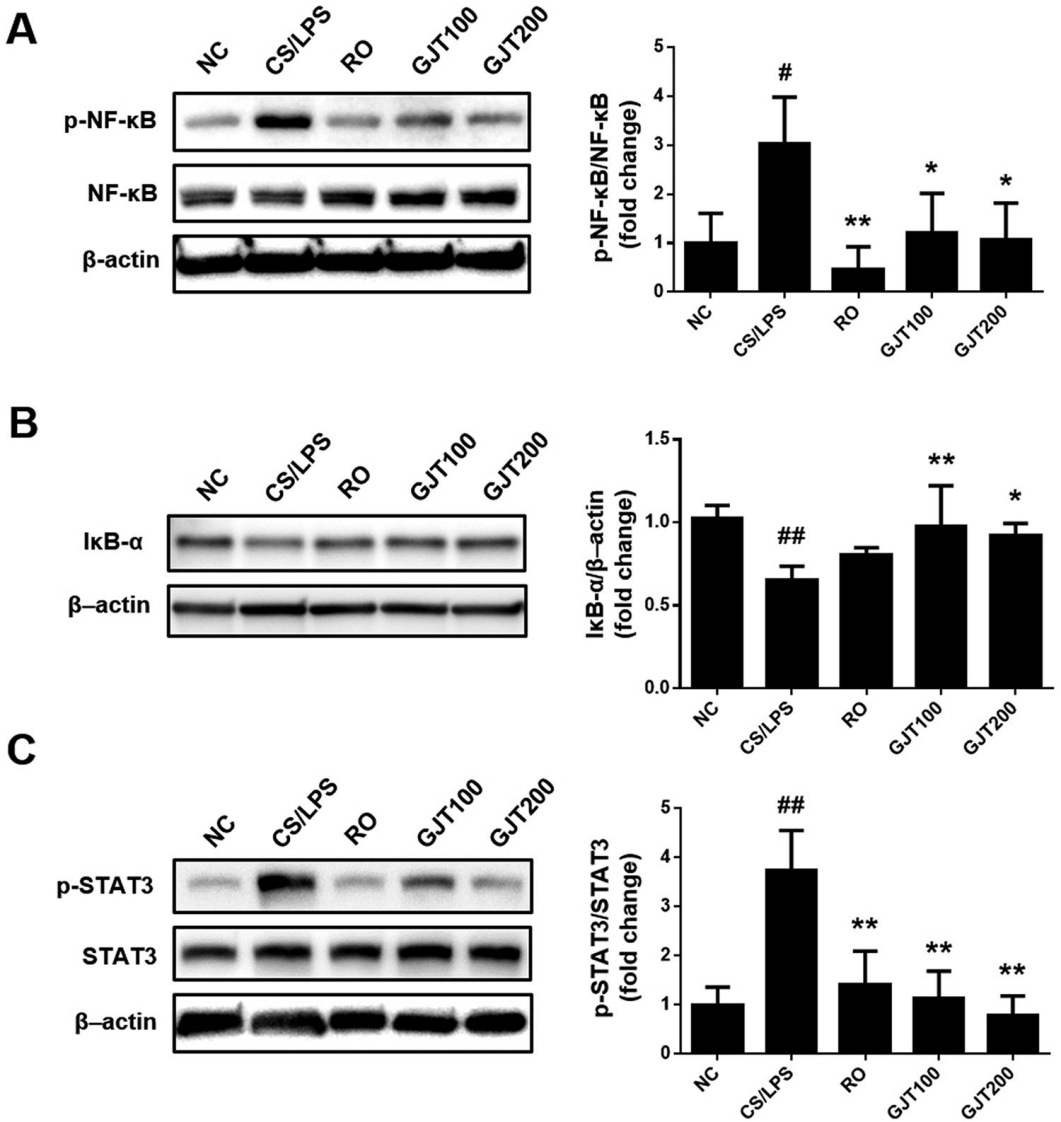
Effects of GJT on CS/LPS-induced/NF-κB and STAT3 pathway activation. Western blot analysis was used to identify the NF-κB (A), IκB-α (B), and STAT3 (C) in lung tissues. NC, normal control; CS/LPS, CS plus LPS-exposed; RO, roflumilast (5 mg/kg) administered prior to CS/LPS exposure; GJT100, GJT (100 mg/kg) administered prior to CS/LPS exposure; GJT200, GJT (200 mg/kg) administered prior to CS/LPS exposure. Results are presented as mean ± SD. ^#^*p* < 0.05 and ^##^*p* < 0.01 compared with the NC group; *p < 0.05 and **p < 0.01 compared with the CS/LPS group.

As the NF-κB and STAT3 pathways were reported to play roles in inflammatory processes, including those of lung disease (Liu et al. 2013; Kasembeli et al. 2018), we examined whether GJT treatment could affect these pathways in our system. Indeed, the phosphorylation of the p65 subunit of NF-κB was markedly higher in lung tissues from CS/LPS-treated mice compared with NC mice, whereas lung tissues from roflumilast- or GJT-treated animals did not show this elevation of p65 NF-κB phosphorylation (Figure 5(A)). Release of NF-κB from the IκB-α-containing complex then translocate to the nucleus to activate the transcription of its target genes (Hayden and Ghosh 2008). To confirm NF-κB activation, we examined IκB-α expression in lung tissues. As shown in Figure 5(B), GJT treatment inhibited IκB-α degradation induced by CS/LPS indicating NF-κB activation. Similarly, the level of phosphorylated STAT3 was elevated in the CS/LPS group relative to the NC group, and this change was reversed in the roflumilast and GJT groups (Figure 5(C)). These results suggest that GJT acts at least partly via NF-κB and STAT3 signalling, and that these pathways may contribute to the anti-inflammatory effects of GJT on airway-derived epithelial inflammation.

### GJT suppresses CS/LPS-induced structural changes in lung tissues

**Figure 6.**
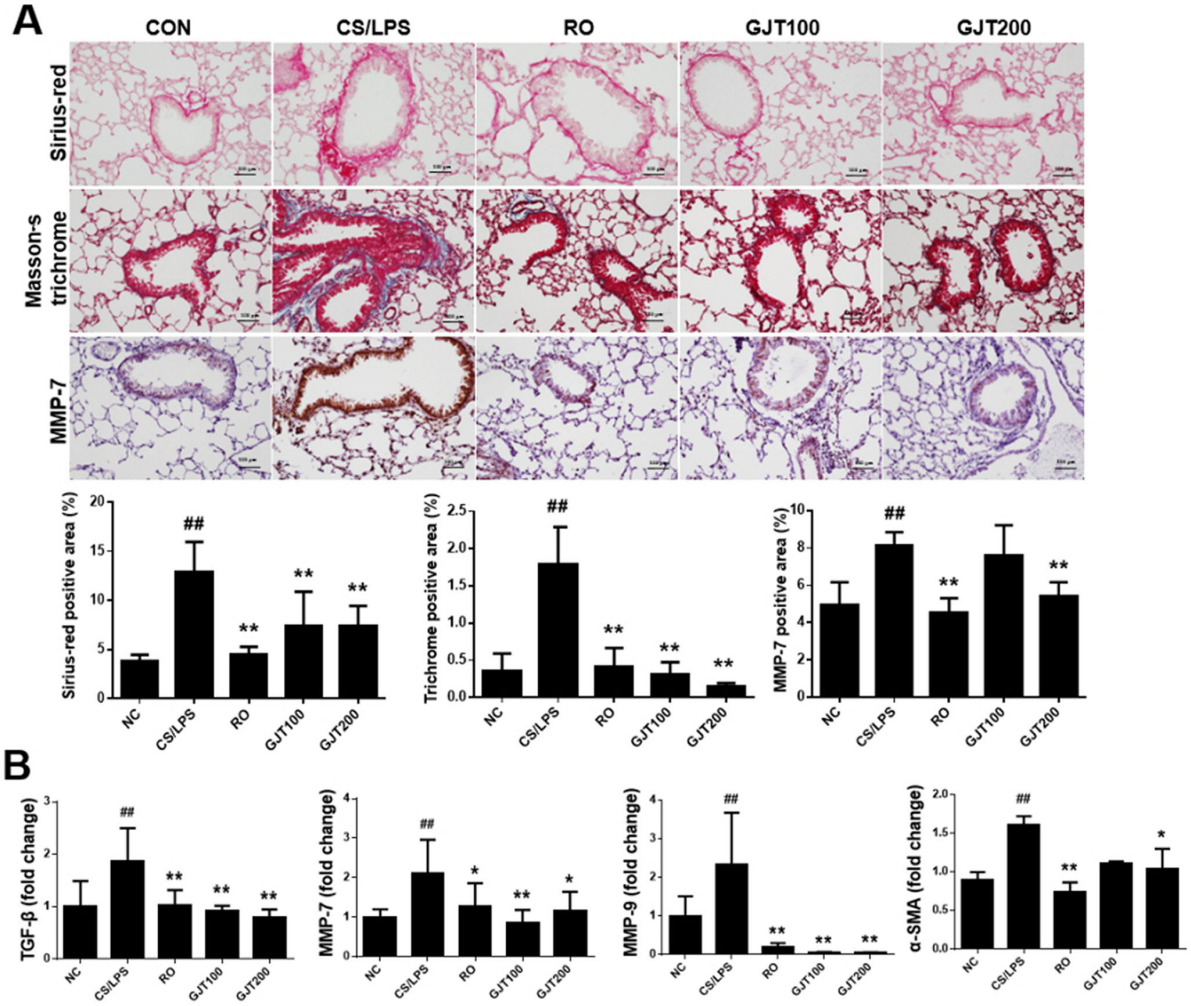
Effects of GJT on CS/LPS-induced structural changes in lung tissues. (A) Sirius-red staining, Masson’s trichrome staining and IHC of MMP-7 (×200 magnification). (B) Relative mRNA expression levels of TGF-β, MMP-7, MMP-9, and α-SMA in lung tissues. NC, normal control; CS/LPS, CS plus LPS-exposed; RO, roflumilast (5 mg/kg) administered prior to CS/LPS exposure; GJT100, GJT (100 mg/kg) administered prior to CS/LPS exposure; GJT200, GJT (200 mg/kg) administered prior to CS/LPS exposure. Results are presented as mean ± SD. ^#^*p* < 0.05 and ^##^*p* < 0.01 compared with the NC group; *p < 0.05 and **p < 0.01 compared with the CS/LPS group.

To investigate the effect of GJT on structural changes and airway remodelling, we performed histological examination and assessed the relative mRNA expression levels of relevant genes. Sirius-red staining and Masson’s trichrome staining showed that there was increased peribronchiolar collagen deposition in the lungs of CS/LPS-exposed mice, and this elevation was significantly alleviated in mice treated with roflumilast or GJT (Figure 6(A)). In addition, the area positive for the structural remodelling marker, MMP-7, was increased in CS/LPS-treated animals compared with NC group, but this elevation was attenuated in roflumilast or GJT-treated animals (Figure 6(A)). Consistently, the relative expression level of the lung fibrosis marker, TGF-β and α-SMA was elevated in the CS/LPS group relative to NC group, but this change was reversed in animals treated with roflumilast or either dose of GJT (Figure 6(B)). Finally, the levels of MMP-7 and MMP-9 were increased in the CS/LPS group compared with the NC group, but these changes were significantly suppressed in mice that received roflumilast or either dose of GJT (Figure 6(B)).

## Discussion

COPD is a chronic inflammatory disease characterized by progressive and irreversible airflow limitation, mucous overproduction, chronic bronchitis, and small-airway remodelling. Although many drugs are currently marketed for COPD, their reported side effects result in low compliance, and therefore new drugs for alternative treatments are needed (Wang et al. 2020). The present study examined the effect of GJT in a mouse model of COPD. Here, we report that treatment with GJT markedly decreases the CS/LPS exposure-induced infiltration of inflammatory cells and the production of proinflammatory cytokines in BALF and lung tissues. It also attenuated CS/LPS-induced airway wall thickening and fibrosis and effectively suppressed the CS/LPS-induced upregulations of TGF-β and MMPs.

The molecular pathology underlying COPD is influenced by the genetic background, cellular status, and chronic inhalation of toxic particles, especially CS. The response to CS involves the innate immune system, which triggers airway epithelial cells and alveolar macrophages to release proinflammatory cytokines and chemokines, such as TNF-α, IL-6, IL-1β, and IL-8. This elicits the expression of adhesion molecules on endothelial cells and the recruitment of neutrophils and inflammatory macrophages to the lungs (Brusselle et al. 2011; Barnes 2016). Among the above-listed factors, TNF-α acts as a master regulator of proinflammatory cytokine production, induces the influx of inflammatory cells into the lungs, and has been associated with pulmonary fibrosis and emphysema in animal models of COPD (Maini et al. 1995; Lundblad et al. 2005). TNF-α also induces the expression of adhesion molecules on endothelial cells, thereby facilitating the migration of various inflammatory cell types (Barnes 2016). In COPD, the Th17/T_reg_ ratios in the blood and sputum are shifted towards Th17 cells, and IL-1β and IL-6 were thought to involve the signalling via Th17 cells (Hikichi et al. 2019). IL-6 is considered to be a key proinflammatory cytokine that plays important roles in the disease progression and tissue injuries of several autoimmune diseases, including multiple sclerosis, rheumatoid arthritis, and systemic lupus erythematosus. IL-6 also importantly modulates CD4 T cell effector functions to produce immune responses and contribute to inflammation (Dienz and Rincon 2009). IL-8, which is also called C-X-C motif chemokine 8 (CXCL-8), is a chemokine known to be involved in inflammation-mediated neutrophil infiltration and chemotaxis (Chung 2001). Previous studies showed that IL-8 is increased in the BALF and sputum of COPD patients, and that the serum IL-8 level is correlated with COPD severity (Chung 2001). In the present study, we show that GJT treatment significantly attenuates CS/LPS-induced inflammatory cell infiltration into lung tissues, reduces the CS/LPS-enhanced inflammatory cell counts (total cells and macrophages) in BALF, and decreases the CS/LPS-induced upregulations of proinflammatory cytokines, including TNF-α, IL-6, IL-1β, and IL-8, in lung tissues. These results show that GJT significantly suppresses the inflammatory response associated with this mouse model of COPD.

NF-κB is known to regulate lung pathology by modulating the expression levels of vital proinflammatory cytokines and chemokines (Liu et al. 2013; Shin et al. 2017). When an extracellular stimulus acts on receptors of the respiratory epithelium, NF-κB (p50 and p65) is translocated into the nucleus, where it binds specific DNA elements to trigger an inflammatory response (Wang et al. 2020). The cytokines expressed under control of the NF-κB pathway play roles in inflammatory cell infiltration and evoke oxidative stress, leading to COPD progression (Adcock et al. 2011). In addition, the JAK/STAT and NF-κB pathways were found to be closely related and STAT3 signalling contributes to the inflammatory response (Wang and Sun 2014; Kasembeli et al. 2018). NF-κB upregulates a variety of proinflammatory mediators, including TNF-α, IL-1α, and IL-6. STAT3 is activated by various molecules, especially IL-6. Phosphorylated STAT3 translocated to the nucleus, where it regulates genes specifically related to inflammation (Kasembeli et al. 2018). Notably, pharmacological drug targeting of STAT3 has been shown to significantly improve inflammatory diseases, including asthma and inflammatory bowel disease (Kasembeli et al. 2018). Recently, Huan et al.’s study (Huan et al. 2017) suggested that ergosterol improved COPD via the JAK3/STAT3/NF-κB pathway in a mouse model of COPD. Here, we report that GJT treatment significantly decreased the CS/LPS-induced phosphorylation of NF-κB and STAT3, indicating that the protective effects of GJT on CS/LPS-induced airway inflammatory responses appear to be closely associated with its ability to downregulate the activation levels of NF-κB and STAT3.

Chronic exposure to CS leads to morphological and functional changes in the airway epithelium. CS-mediated ROS release has been shown to disrupt the tight junctions of airway epithelial cells via an epidermal growth factor receptor-dependent mechanism (Petecchia et al. 2009). The disruption of the epithelial barrier and a concomitant downregulation of E-cadherin induces the epithelial–mesenchymal transition, which leads to the overproduction of MMPs and growth factors, destruction of airways, and remodelling of lung tissues (Milara et al. 2013). Furthermore, alveolar macrophages produce ROS and MMPs to disrupt alveolar structures and induce fibrotic mediators, such as TGF-β, to trigger airway remodelling. In patients with COPD, MMP-7 and MMP-9 are upregulated in various samples, including serum, plasma, sputum, BALF, and lung tissues (Navratilova et al. 2012; Montaño et al. 2014; Kraen et al. 2019). MMP-7 and MMP-9 can disrupt the normal alveolar architecture, increase inflammatory responses in lung tissues, and trigger emphysema, collectively leading to loss of lung function (Churg et al. 2012; Shin et al. 2014). Meanwhile, TGF-β is a lung fibrosis-related cytokine that can be released by various cells, such as macrophages, epithelial cells, and fibroblasts (Sutliff et al. 2010; Chen et al. 2016). In the current study, we found that GJT treatment attenuated the CS/LPS-induced increases in the collagen accumulation of bronchioles, thickening of alveolar walls, and expression levels of MMP-7, MMP-9, and TGF-β, indicating that GJT ameliorates this mouse model of COPD at least in part by suppressing airway remodelling.

## Conclusions

We show that GJT exerts a protective effect against LPS- and CS-induced COPD in a mouse model by reducing inflammatory responses, airway remodelling, NF-κB activity, and STAT3 activity. Therefore, GJT may be potential therapy for the treatment of COPD.

## References

[CIT0001] Adcock IM, Caramori G, Barnes PJ. 2011. Chronic obstructive pulmonary disease and lung cancer: new molecular insights. Respiration. 81(4):265–284. 21430413 10.1159/000324601

[CIT0002] Barnes PJ. 2016. Inflammatory mechanisms in patients with chronic obstructive pulmonary disease. J Allergy Clin Immunol. 138(1):16–27. 27373322 10.1016/j.jaci.2016.05.011

[CIT0003] Bourdin A, Burgel PR, Chanez P, Garcia G, Perez T, Roche N. 2009. Recent advances in COPD: pathophysiology, respiratory physiology and clinical aspects, including comorbidities. Eur Respir Rev. 18(114):198–212. 20956145 10.1183/09059180.00005509

[CIT0004] Brusselle GG, Joos GF, Bracke KR. 2011. New insights into the immunology of chronic obstructive pulmonary disease. Lancet. 378(9795):1015–1026. 21907865 10.1016/S0140-6736(11)60988-4

[CIT0005] Chen J, Yang X, Zhang W, Peng D, Xia Y, Lu Y, Han X, Song G, Zhu J, Liu R. 2016. Therapeutic effects of resveratrol in a mouse model of LPS and cigarette smoke-Induced COPD. Inflammation. 39(6):1949–1959. 27590234 10.1007/s10753-016-0430-3

[CIT0006] Chung KF. 2001. Cytokines in chronic obstructive pulmonary disease. Eur Respir J Suppl. 34:50s–59s. 12392035

[CIT0007] Churg A, Zhou S, Wright JL. 2012. Series "matrix metalloproteinases in lung health and disease": Matrix metalloproteinases in COPD. Eur Respir J. 39(1):197–209. 21920892 10.1183/09031936.00121611

[CIT0008] Delneste Y, Beauvillain C, Jeannin P. 2007. Innate immunity: structure and function of TLRs. Med Sci. 23(1):67–73 [in French]. 10.1051/medsci/200723167 17212934

[CIT0009] Dienz O, Rincon M. 2009. The effects of IL-6 on CD4 T cell responses. Clin Immunol. 130(1):27–33. 18845487 10.1016/j.clim.2008.08.018 PMC2660866

[CIT0010] Garnock-Jones KP. 2015. Roflumilast: a review in COPD. Drugs. 75(14):1645–1656. 26338438 10.1007/s40265-015-0463-1

[CIT0011] Gori S, Alcain J, Vanzulli S, Moreno Ayala MA, Candolfi M, Jancic C, Geffner J, Vermeulen M, Salamone G. 2019. Acetylcholine-treated murine dendritic cells promote inflammatory lung injury. PLoS One. 14(3):e0212911. 30822345 10.1371/journal.pone.0212911 PMC6396899

[CIT0012] Hayden MS, Ghosh S. 2008. Shared principles in NF-kappaB signaling. Cell. 132(3):344–362. 18267068 10.1016/j.cell.2008.01.020

[CIT0013] Hikichi M, Mizumura K, Maruoka S, Gon Y. 2019. Pathogenesis of chronic obstructive pulmonary disease (COPD) induced by cigarette smoke. J Thorac Dis. 11(Suppl 17):S2129–S2140. 31737341 10.21037/jtd.2019.10.43 PMC6831915

[CIT0014] Hillmer EJ, Zhang H, Li HS, Watowich SS. 2016. STAT3 signaling in immunity. Cytokine Growth Factor Rev. 31:1–15. 27185365 10.1016/j.cytogfr.2016.05.001 PMC5050093

[CIT0015] Huan W, Tianzhu Z, Yu L, Shumin W. 2017. Effects of ergosterol on COPD in mice via JAK3/STAT3/NF-kappaB pathway. Inflammation. 40(3):884–893. 28251448 10.1007/s10753-017-0533-5

[CIT0016] Heo J. 2004. Donguibogam: principles and practice of eastern medicine. Republic of Korea: Namsandang.

[CIT0017] Lim JS, Hahn D, Gu MJ, Oh J, Lee JS, Kim J-S. 2019. Anti-inflammatory and antioxidant effects of 2,7-dihydroxy-4, 6-dimethoxy phenanthrene isolated from *Dioscorea batatas* Decne. Appl Biol Chem. 62(1):29.

[CIT0018] Jung HW, Yoon CH, Park KM, Han HS, Park YK. 2009. Hexane fraction of Zingiberis Rhizoma Crudus extract inhibits the production of nitric oxide and proinflammatory cytokines in LPS-stimulated BV2 microglial cells via the NF-kappaB pathway. Food Chem Toxicol. 47(6):1190–1197. 19233241 10.1016/j.fct.2009.02.012

[CIT0019] Kasembeli MM, Bharadwaj U, Robinson P, Tweardy DJ. 2018. Contribution of STAT3 to inflammatory and fibrotic diseases and prospects for its targeting for treatment. IJMS. 19(8):2299–2328. 10.3390/ijms19082299 PMC6121470 30081609

[CIT0020] Kraen M, Frantz S, Nihlen U, Engstrom G, Lofdahl CG, Wollmer P, Dencker M. 2019. Matrix metalloproteinases in COPD and atherosclerosis with emphasis on the effects of smoking. PLoS One. 14(2):e0211987. 30789935 10.1371/journal.pone.0211987 PMC6383934

[CIT0021] Lane N, Robins RA, Corne J, Fairclough L. 2010. Regulation in chronic obstructive pulmonary disease: the role of regulatory T-cells and Th17 cells. Clin Sci (Lond). 119(2):75–86. 20402669 10.1042/CS20100033

[CIT0022] Liu R, Bai J, Xu G, Xuan L, Zhang T, Meng A, Hou Q. 2013. Multi-allergen challenge stimulates steriod-resistant airway inflammation via NF-kappaB-mediated IL-8 expression. Inflammation. 36(4):845–854. 23456484 10.1007/s10753-013-9611-5

[CIT0023] Lundblad LK, Thompson-Figueroa J, Leclair T, Sullivan MJ, Poynter ME, Irvin CG, Bates JH. 2005. Tumor necrosis factor-alpha overexpression in lung disease: a single cause behind a complex phenotype. Am J Respir Crit Care Med. 171(12):1363–1370. 15805183 10.1164/rccm.200410-1349OC PMC2718479

[CIT0024] Maini RN, Elliott MJ, Brennan FM, Feldmann M. 1995. Beneficial effects of tumour necrosis factor-alpha (TNF-alpha) blockade in rheumatoid arthritis (RA). Clin Exp Immunol. 101(2):207–212. 7648705 10.1111/j.1365-2249.1995.tb08340.x PMC1553280

[CIT0025] Metcalfe HJ, Lea S, Hughes D, Khalaf R, Abbott-Banner K, Singh D. 2014. Effects of cigarette smoke on Toll-like receptor (TLR) activation of chronic obstructive pulmonary disease (COPD) macrophages. Clin Exp Immunol. 176(3):461–472. 24528166 10.1111/cei.12289 PMC4008991

[CIT0026] Milara J, Peiro T, Serrano A, Cortijo J. 2013. Epithelial to mesenchymal transition is increased in patients with COPD and induced by cigarette smoke. Thorax. 68(5):410–420. 23299965 10.1136/thoraxjnl-2012-201761

[CIT0027] Moldoveanu B, Otmishi P, Jani P, Walker J, Sarmiento X, Guardiola J, Saad M, Yu J. 2009. Inflammatory mechanisms in the lung. J Inflamm Res. 2:1–11. 22096348 PMC3218724

[CIT0028] Montaño M, Sansores RH, Becerril C, Cisneros J, González-Avila G, Sommer B, Ochoa L, Herrera I, Ramírez-Venegas A, Ramos C. 2014. FEV1 inversely correlates with metalloproteinases 1, 7, 9 and CRP in COPD by biomass smoke exposure. Respir Res. 15(1):74 10.1186/1465-9921-15-74 PMC4086695 24980707

[CIT0029] Navratilova Z, Zatloukal J, Kriegova E, Kolek V, Petrek M. 2012. Simultaneous up-regulation of matrix metalloproteinases 1, 2, 3, 7, 8, 9 and tissue inhibitors of metalloproteinases 1, 4 in serum of patients with chronic obstructive pulmonary disease. Respirology. 17(6):1006–1012. 22591289 10.1111/j.1440-1843.2012.02197.x

[CIT0030] Pastorino G, Cornara L, Soares S, Rodrigues F, Oliveira M. 2018. Liquorice (*Glycyrrhiza glabra*): a phytochemical and pharmacological review. Phytother Res. 32(12):2323–2339. 30117204 10.1002/ptr.6178 PMC7167772

[CIT0031] Petecchia L, Sabatini F, Varesio L, Camoirano A, Usai C, Pezzolo A, Rossi GA. 2009. Bronchial airway epithelial cell damage following exposure to cigarette smoke includes disassembly of tight junction components mediated by the extracellular signal-regulated kinase 1/2 pathway. Chest. 135(6):1502–1512. 19447922 10.1378/chest.08-1780

[CIT0032] Saklatvala J, Dean J, Clark A. 2003. Control of the expression of inflammatory response genes. Biochem Soc Symp. 70:95–106. 10.1042/bss0700095 14587285

[CIT0033] Shin IS, Park JW, Shin NR, Jeon CM, Kwon OK, Kim JS, Kim JC, Oh SR, Ahn KS. 2014. Melatonin reduces airway inflammation in ovalbumin-induced asthma. Immunobiology. 219(12):901–908. 25161126 10.1016/j.imbio.2014.08.004

[CIT0034] Shin NR, Kim SH, Ko JW, Park SH, Lee IC, Ryu JM, Kim JC, Shin IS. 2017. HemoHIM, a herbal preparation, alleviates airway inflammation caused by cigarette smoke and lipopolysaccharide. Lab Anim Res. 33(1):40–47. 28400838 10.5625/lar.2017.33.1.40 PMC5385281

[CIT0035] Sutliff RL, Kang BY, Hart CM. 2010. PPARgamma as a potential therapeutic target in pulmonary hypertension. Ther Adv Respir Dis. 4(3):143–160. 20530063 10.1177/1753465809369619 PMC3978142

[CIT0036] Tamaoki J, Kondo M, Tagaya E, Takemura K, Konno K. 1996. Zizyphi fructus, a constituent of antiasthmatic herbal medicine, stimulates airway epithelial ciliary motility through nitric oxide generation. Exp Lung Res. 22(3):255–266. 8792120 10.3109/01902149609031774

[CIT0037] Wang C, Zhou J, Wang J, Li S, Fukunaga A, Yodoi J, Tian H. 2020. Progress in the mechanism and targeted drug therapy for COPD. Signal Transduct Target Ther. 5(1):248. 33110061 10.1038/s41392-020-00345-x PMC7588592

[CIT0038] Wang SW, Sun YM. 2014. The IL-6/JAK/STAT3 pathway: potential therapeutic strategies in treating colorectal cancer (Review). Int J Oncol. 44(4):1032–1040. 24430672 10.3892/ijo.2014.2259

[CIT0039] Yang H, Cheng X, Yang YL, Wang YH, Du GH. 2017. Ramulus Cinnamomi extract attenuates neuroinflammatory responses via downregulating TLR4/MyD88 signaling pathway in BV2 cells. Neural Regen Res. 12(11):1860–1864. 29239332 10.4103/1673-5374.219048 PMC5745840

[CIT0040] Yoo SR, Kim Y, Lee MY, Kim OS, Seo CS, Shin HK, Jeong SJ. 2016. Gyeji-tang water extract exerts anti-inflammatory activity through inhibition of ERK and NF-kappaB pathways in lipopolysaccharide-stimulated RAW 264.7 cells. BMC Complement Altern Med. 16(1):390. 27733198 10.1186/s12906-016-1366-8 PMC5062814

